# Generation of a three-dimensional collagen scaffold-based model of the human endometrium

**DOI:** 10.1098/rsfs.2019.0079

**Published:** 2020-02-14

**Authors:** Yassen Abbas, Lucia G. Brunel, Michael S. Hollinshead, Ridma C. Fernando, Lucy Gardner, Imogen Duncan, Ashley Moffett, Serena Best, Margherita Y. Turco, Graham J. Burton, Ruth E. Cameron

**Affiliations:** 1 Department of Pathology, University of Cambridge, Cambridge CB2 1QP, UK; 2 Centre for Trophoblast Research (CTR), University of Cambridge, Cambridge CB2 3EG, UK; 3 Department of Physiology, Development and Neuroscience, University of Cambridge, Cambridge CB2 3EG, UK; 4 Department of Materials Science and Metallurgy, University of Cambridge, 27 Charles Babbage Road, Cambridge CB3 0FS, UK

**Keywords:** collagen scaffolds, organoids, endometrium, co-culture

## Abstract

The endometrium is the secretory lining of the uterus that undergoes dynamic changes throughout the menstrual cycle in preparation for implantation and a pregnancy. Recently, endometrial organoids (EO) were established to study the glandular epithelium. We have built upon this advance and developed a multi-cellular model containing both endometrial stromal and epithelial cells. We use porous collagen scaffolds produced with controlled lyophilization to direct cellular organization, integrating organoids with primary isolates of stromal cells. The internal pore structure of the scaffold was optimized for stromal cell culture in a systematic study, finding an optimal average pore size of 101 µm. EO seeded organize to form a luminal-like epithelial layer, on the surface of the scaffold. The cells polarize with their apical surface carrying microvilli and cilia that face the pore cavities and their basal surface attaching to the scaffold with the formation of extracellular matrix proteins. Both cell types are hormone responsive on the scaffold, with hormone stimulation resulting in epithelial differentiation and stromal decidualization.

## 1. Introduction

**Figure 1.**
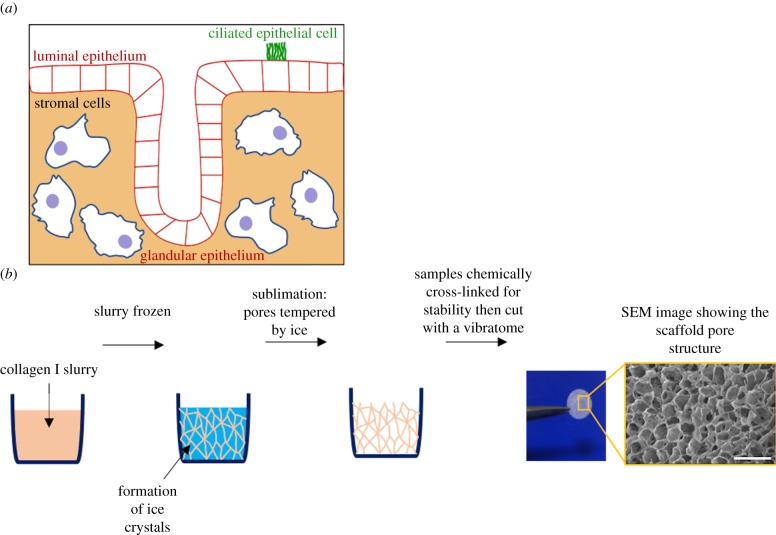
Porous collagen scaffolds are used to develop a model of the human endometrium. (*a*) Schematic of the human endometrium comprising epithelial and stromal cells. There are two epithelial cell populations, the luminal that lines the lumen of the uterus and glandular epithelium, which secrete factors required to maintain the conceptus pre- and post-implantation. Epithelial cells are polarized, with the apical surface facing the outside surface and basal inwards. A number of epithelial cells are ciliated. (*b*) Porous collagen scaffolds used to develop a model of the endometrium are produced by lyophilization. An aqueous slurry of collagen I is frozen. As ice crystals form, the solid material in the slurry is pushed to the boundaries of the ice crystals. The pressure of the chamber is then lowered, and the temperature is raised, inducing sublimation of the ice crystals. The final product is a porous scaffold with solid material lining the spaces previously occupied by the ice crystals. Samples are cross-linked for stability then cut with a vibratome. Scale bar, 400 µm.

The endometrium is the inner mucosal lining of the uterus, which undergoes dynamic changes of shedding, regeneration and differentiation under the control of ovarian hormones during the menstrual cycle. It is composed of a surface (luminal) epithelium with glands that invaginate into the stroma containing stromal, immune and endothelial cells (figure 1*a*).

The endometrium is essential for a successful pregnancy as it is the site of implantation (luminal epithelium) and subsequent nutrition of the developing conceptus (glandular epithelium) [1]. The proper function of the endometrium depends on its efficient hormonal response and interactions among the different cell types. Endometrial dysfunction results in a range of disorders from abnormal bleeding to infertility and pregnancy failure [2,3]. However, the nature of these cellular interactions is still largely undefined in humans.

Endometrial epithelial cells are characterized by their apical–basal polarity, tight junctions between the cells and can be identified with immunostaining for epithelial cell adhesion molecule (EPCAM). At the apical surface, microvilli and ciliated cells face the lumen of a gland or the uterine cavity, whereas the basal surface is attached to a specialized extracellular matrix (ECM) called the basement membrane [4,5]. Endometrial stromal cells are fibroblasts of mesenchymal origin, and are commonly identified with the maker vimentin [6]. The cell–cell communication between epithelial and stromal cells is important for normal endometrial function and maintenance of pregnancy. Molecular cues, which are produced under the action of oestrogen and progesterone and result in differentiation and decidualization of epithelial and stromal cells, respectively, are exchanged between these cells types [7]. The recent derivation of long-term, hormone-responsive organoids of the endometrial epithelium has enabled proliferative epithelial progenitor cells to be isolated from the human endometrium for the first time [5]. However, these organoids lack the stromal component and are inverted, in that the apical cell membrane is directed towards a central cavity. Thus, there is a need for an *in vitro* model that recapitulates the epithelial and stromal components of the endometrium in order to study endometrial function.

We set out to build upon the recently established organoid system and develop a co-culture model of human endometrial epithelial and stromal cells using a bioengineering approach [5]. Previously, several methodologies were taken such as co-culture within a hydrogel, endometrial explants and culture on decellularized endometrial tissue [8–10]. However, these models do not fully recapitulate the intricate tissue architecture of the endometrium and reproducibility is limited. Thus, we took an alternative approach by developing three-dimensional porous collagen scaffolds, tailored for seeding the two main endometrial cell types. Scaffolds have many potential advantages as a substrate since they: (i) provide a structural support for cells to adhere to, (ii) are a permissive environment that enables cells to grow and produce their own ECM, (iii) allow efficient dissolved gas and nutrient exchange due to the presence of pores, and (iv) facilitate handling of the sample [11–13]. We have previously used collagen scaffolds to study cell migration in breast cancer and to model the mammary gland [14–18].

In this work, collagen scaffolds are generated by lyophilization, as previously described [15]. Briefly, an aqueous slurry of bovine collagen I is frozen to form ice crystals that are subsequently removed by sublimation, leaving a porous structure with solid material lining (figure 1*b*). Factors such as pore size and interconnectivity can be optimized for different cell types and tissue applications and this is done by adjusting select parameters of the lyophilization process [19,20]. The length of time the slurry spends at the equilibrium temperature while ice crystals actively grow is one parameter in the thermal profile of the slurry that can be adjusted to control scaffold pore size [19,21]. For example, the material and shape of the moulds in which the scaffolds are produced affect the amount of time that the slurry spends at equilibrium [22,23]. Moulds with higher thermal conductivities cause the slurry to have faster cooling and nucleation rates, influencing the time at equilibrium of the ice crystals and thereby changing the pore size. For moulds made of the same material, heat transfer through the slurry is faster for a mould of a smaller diameter. In addition to pore size, pore interconnectivity is another important feature that contributes to the ability of a scaffold to support cell migration and molecular transport. The percolation diameter is a characteristic that encompasses both pore size and interconnectivity, and it is determined by taking measurements of the distances that virtual spheres of different diameters are able to travel through the scaffold [20,24,25].

In an attempt to mimic the structure of the endometrium and incorporate the endometrial stromal cells, a three-dimensional porous collagen scaffold was designed to direct the cellular organization of both stromal and epithelial cells. Endometrial organoids (EO) seeded form a luminal-like epithelial layer on the scaffold, with apical polarization towards the outside surface. Both stromal and epithelial cells are functionally responsive to hormones and produce their own matrix proteins on the scaffold, thus providing a co-culture model as a basis for a more complex model of the endometrium.

## 2. Material and methods

### 2.1. Collagen scaffold fabrication

A suspension of 1% (w/v) insoluble, type I collagen (Collagen Solutions, UK) derived from bovine dermal collagen was prepared by hydrating the collagen in 0.05 M acetic acid (Sigma-Aldrich, UK) for at least 48 h at 4°C. The suspension was blended to generate a uniform slurry and centrifuged for 5 min at 2500 rpm to remove air bubbles formed during blending. The slurry was pipetted into moulds before lyophilization: (i) a 316L stainless steel cylindrical well with a diameter of 46.5 mm, (ii) a polystyrene 48-well cell culture plate, and (iii) a polystyrene 6-well cell culture plate. The collagen slurries were frozen in a VirTis AdVantage freeze-dryer (Biopharma Process Systems, UK) that was ramped from room temperature to −30°C and held for 3 h. The slurries were subsequently sublimed at 0°C for 20 h under a vacuum of less than 100 mTorr.

The lyophilized collagen scaffolds were chemically cross-linked using carbodiimide at 30% of the molar ratio developed by Olde Damink *et al*., which is 5 : 2 : 1 of 1-ethyl-3-(3-dimethylaminopropyl)-carbodiimide hydrochloride (Sigma-Aldrich, UK) and *N*-hydroxysuccinimide (Sigma-Aldrich, UK) relative to the collagen carboxylic acid group [26,27]. The cross-linking solution was prepared in 95% (v/v) ethanol and made freshly before use. The scaffolds were submerged in the cross-linking solution for 2 h at room temperature. After cross-linking, the scaffolds were washed in distilled water (5 × 5 min). The scaffolds were then frozen at −30°C and lyophilized again in their moulds using the previously described freezing and drying cycle.

### 2.2. Scaffold characterization

Scanning electron microscopy (SEM) was conducted to view the surface morphology of pores within the scaffold using a Nova NanoSEM (FEI Company, The Netherlands) in secondary electron imaging mode operated at 5 kV with a spot size of 3.0 nm. Samples were prepared for SEM by cutting cross-sections in the centre of the scaffolds with a scalpel. The scaffold samples were mounted onto a metallic stub with carbon tape and sputter-coated in an Emitech K550 (Emitech, UK) with gold for 2–3 min at a current of 20 mA.

The three-dimensional inner pore structure of produced scaffolds was visualized by micro-computed tomography (micro-CT) using a Skyscan 1172 X-ray mictrotomograph (Bruker, Belgium). Scaffolds were cut with an 8 mm biopsy punch and placed on a sample holder in the micro-CT. Before scanning, a flat field correction was applied to remove artefacts from the images caused by variations in the pixel-to-pixel sensitivity of the detector or distortions in the optical path. An operating voltage of 25 kV, current of 138 µA and pixel size of 2.97 µm were used for the scans. The projection data from the micro-CT were reconstructed using NRecon, a software program included in the Skyscan package.

The micro-CT reconstructions of the scaffolds were used to determine their pore size and percolation diameter as previously described by Ashworth *et al*. Briefly, the pore sizes were analysed with a three-step process on CTAn: the automatic Otsu method for thresholding, the sweep function for despeckling to remove noise and the three-dimensional analysis function for calculating pore size [20]. For the percolation diameter, which is defined as the diameter of the largest spherical object able to penetrate through an infinitely large scaffold, the ‘shrink wrap’ feature used by Ashworth *et al*. [20] was used to identify the volume accessible to the virtual sphere. The diameter *d* of this sphere was progressively increased so that the corresponding length of the volume accessible to the object *L* could be measured according to the following equation from percolation theory: *L* = *L_o_*(*d* − *d_c_*)^−^*^v^*_,_ where *v* is 0.88 for a three-dimensional system. The percolation diameter *d_c_* was obtained by finding the intercept of the plot of *d* as a function of *L*^−1/*v*^.

### 2.3. Isolation of stromal cells and endometrial organoids

Samples of decidual tissue were taken from routine terminations of pregnancy (6–12 weeks' gestation) as previously described for the isolation of decidual stromal cells [28]. Ethical approval for sampling decidua was obtained from the Cambridgeshire Research Committee (reference no. 04/Q0108/23) and East of England Cambridge Central Research Ethics Committee (reference no. 17/EE/0151 IRAS 225205). Cells were used for experiments only after passage 2, to enable the stroma to de-decidualize. Stromal cells were cultured in Advanced DMEM/F-12 (Thermo Fisher Scientific, USA) supplemented with 10% (v/v) charcoal-stripped fetal bovine serum (FBS; Sigma, USA), 2 mM l^−1^ glutamine, 10 units ml^−1^ penicillin, 100 µg ml^−1^ streptomycin and 2 mg ml^−1^ gentamicin (Thermo Fisher Scientific, USA). Culture medium was replaced every 2–3 days. One week after plating, cells were removed from tissue culture flasks with 0.2% trypsin to be either passaged at a ratio of 1:3 or seeded onto scaffolds. Medium was replaced every 2–3 days.

EO were derived from biopsies of endometrium at the secretory phase (6 and 10 days after pre-ovulatory luteinizing hormone surge) of the mensural cycle, from the Bourn Hall Fertility Clinic with ethical approval from East of England Cambridge Central Research Ethics Committee (reference no. 17/EE/0151 IRAS 225205) and as previously described [5]. Isolated tissue fragments were embedded in Matrigel^®^ (Corning, USA) and cultured in Advanced DMEM/F-12 supplemented with 1× N2 supplement, 1× B27 supplement minus vitamin A (ThermoFisher, USA), 100 µg ml^−1^ Primocin (Invivogen, USA), 1.25 mM *N*-Acetyl-l-cysteine (Sigma), 2 mM l-glutamine (ThermoFisher, USA), 50 ng ml^−1^ recombinant human EGF, 100 ng ml^−1^ recombinant human Noggin, 500 ng ml^−1^ recombinant human Rspondin-1 (Peprotech, USA), 100 ng ml^−1^ recombinant human FGF-10, 50 ng ml^−1^ recombinant human HGF (Peprotech, USA), 500 nM ALK-4, -5, -7 inhibitor, A83-01 (System Biosciences, USA) and 10 nM nicotinamide (Sigma). Cultures were passaged every 7–10 days by electronic and manual pipetting at a ratio of 1 : 4 and medium replaced every 2–3 days.

### 2.4. Stromal cells and endometrial organoids scaffold seeding

The scaffolds were cut with an 8 mm biopsy punch and sectioned into 750 µm thick slices using a vibratome (Leica Microsystems, Germany). For stromal cell-only scaffolds, scaffolds were sterilized in ethanol (70% ethanol followed by 100% ethanol) and dried under vacuum within a Stericup^®^ filter unit (Merck Millipore, USA). Each scaffold was submerged in one non-protein binding Eppendorf (Eppendorf, Germany) tube in 250 µl Advanced DMEM/F-12 containing 500 k stromal cells and incubated at 37°C for 45 min. The stromal cell suspension was then pipetted several times to disrupt the cells that had settled and placed in a hybridizer oven (Techne, UK) at 37°C for 45 min for continuous rotation. The scaffolds were transferred to a 24-well plate, with one scaffold per well and covered with 1 ml of stromal cell medium, which was replaced every 2–3 days.

For seeding EO-only scaffolds, scaffolds were sterilized with 70% ethanol, then washed with sterile deionized water and phosphate-buffered saline (PBS) for 15 min. Subsequently, scaffolds were placed in an incubator at 37°C in Advanced DMEM/F-12 for 30 min before seeding. EO fragments were generated by taking two Matrigel^®^ droplets that were confluent after 7–10 days of culture and mechanically disrupting the organoids using an electronic pipette (Xplorer Plus, Eppendorf, Germany) 500 times through a small-bore pipette tip and manually, 60 times. The sizes of 495 organoid fragments were measured manually with Fiji, an open-source imaging software [29]. The images used for measurement were taken on a light microscope (EVOS, Thermo Fisher Scientific) immediately after the organoids were broken into fragments. The majority of the fragments were less than 50 µm and this was seeded on a scaffold with a surface area of 0.5 cm^2^. Fragments were resuspended in 45 µl of Advanced DMEM/F-12 and 20 µl was pipetted on the centre of each scaffold and placed in an incubator for 45 min, after which this was repeated once more. Scaffolds were then carefully transferred into a new well with 1 ml of EO medium, which was replaced every 2–3 days.

For the co-culture experiments, scaffolds were first seeded with stromal cells as described above and incubated for 2 days. Subsequently, scaffolds were washed with Advanced DMEM/F-12 several times for 15 min in order to remove serum proteins. EO fragments were seeded as described above and a volume of 1.0 ml of EO medium added, for culture up to 10 days.

### 2.5. Hormone stimulation of cells on scaffold

For the differentiation of both stromal-only and EO-only scaffolds, 10 nM β-oestradiol (E2, Sigma) was added 2 days post-cell seeding. For stromal cells only, at days 4 and 6, medium was replaced with the following conditions: 10 nM E2 + 1 μM progesterone (P4, Sigma) + 1 μM 8-bromoadenosine 3′,5′-cyclic monophosphate (cAMP, Sigma). For EO-only scaffolds, 20 ng ml^−1^ prolactin (PRL, Peprotech, UK) was added in addition at days 4 and 6 to mimic signals from decidualized stromal cells. Supernatant was collected at days 2, 4, 6 and 8 and frozen at −20°C for storage before downstream analysis.

Enzyme-linked immunosorbent assays (ELISAs) were used to measure protein concentrations of key markers of stromal decidualization (prolactin, a hormone secreted by decidual stromal cells) and epithelial differentiation (glycodelin, one of the principal components of the endometrial glands) in the supernatant. ELISAs for prolactin (DY682, R&Dsystems, USA) and glycodelin (ELH-PP14, Raybiotech, USA) were measured from stromal-only and EO-only scaffolds, respectively. A volume of 100 μl of supernatant was used with each sample measured in duplicate, as per the manufacturer's instructions.

### 2.6. Immunofluorescence and confocal microscopy

Scaffolds were washed with Advanced DMEM/F-12 twice for 10 min before being fixed with 4% (v/v) paraformaldehyde (PFA, Sigma) in PBS for 15 min at room temperature. Samples were washed several times with PBS and cells permeabilized with 0.5% Triton X-100 (Sigma) in PBS for 20 min then washed again in PBS. Cells were blocked with 1% (w/v) bovine serum albumin (Sigma) in PBS with 5% (v/v) goat serum (Sigma) at room temperature for 45 min, in order to prevent unspecific antibody binding. The following primary antibodies were incubated in PBS for 30 min at room temperature: EPCAM (1/100, 2929 and 36746, CellSignalling, USA), vimentin (1/150, V6630, Sigma), collagen I, II, III, IV and V (1/100, ab24117, Abcam, UK) and the marker used to identify cilia, acetyl-α-tubulin (1/100, 12152, CellSignalling, USA). The antibody for the tight-junction protein ZO-1 (1/100, 13663, CellSignalling, USA) was incubated at 4°C overnight. Scaffolds were washed several times with PBS and isotype-specific secondary antibodies (A21202 and A11035, Thermo Fisher Scientific) were applied, at 1/400 in PBS for 1.5 h at room temperature. Scaffolds were washed once more in PBS and incubated with Hoechst nuclear stain (66249, Thermo Fisher Scientific) for a minimum of 15 min at room temperature. They were then transferred onto a 35 mm ibidi glass-bottom dish (81156, ibidi, Germany) and imaged using a Zeiss 700 confocal microscope (Zeiss, Germany) and ZEN microscope software (Zeiss, Germany).

### 2.7. Stromal cell characterization within scaffold

The effect of scaffold pore size on stromal cell density at the scaffold surface and throughout the cross-section was determined. Stromal cells were seeded as described in §2.3, and after 7 days of culture, they were fixed and stained with Hoechst nuclear stain, as described in §2.6. Scaffolds were imaged using a Zeiss 700 confocal microscope to visualize the cell nuclei on the scaffold surfaces and in cross-sections cut through the middle of the scaffolds with a sharp blade. Four images were taken of each scaffold, two from each surface, with an area of 1.417 mm × 1.417 mm and depth of 100 µm. One image of the cell nuclei was taken for each scaffold cross-section. To calculate the cell density at the top 100 µm of the scaffold surfaces, Fiji's automatic particle analyser and the Fiji plugin TrackMate were used for cell counting. To determine the location of cells within the scaffold cross-sections, TrackMate was used for the coordinates of cell nuclei.

### 2.8. Electron microscopy of cells on the scaffold

For transmission electron microscopy (TEM), scaffolds were washed with Advanced DMEM/F-12 twice for 10 min before being fixed in 2 ml of 0.5% glutaraldehyde (Agar Scientific, UK) in 0.2 M sodium cacodylate buffer (pH 7.2, Sigma) for 2 h at room temperature. Samples were then washed in sodium cacodylate buffer, treated with reduced osmium tetroxide 1% (Oxchem, UK), 1.5% potassium ferricyanide (Sigma) at room temperature for 60 min, washed in water, treated with 0.5% magnesium uranyl acetate (Agar Scientific, UK) at 4 °C for 16 h, dehydrated with ethanol rinsed in propylene oxide (Serva, Germany) and embedded in Epon resin (Serva, Germany). Ultrathin sections were examined in an FEI Tecnai G2 TEM at 80 kV (FEI/ Thermo Fisher Scientific). Images were acquired with a MegaView III CCD and Soft Imaging Systems program.

For SEM, scaffolds were rinsed twice in PBS and fixed in 2% (v/v) glutaraldehyde (TAAB, UK)/2% formaldehyde (Merck, USA) in 0.05 M sodium cacodylate buffer (pH 7.4, Sigma) containing 2 mM CaCl_2_ (Sigma) for 3 days at 4°C. The scaffolds were then cut in half with a razor blade and rinsed in deionized water and treated with 1% osmium tetroxide (Oxchem, UK)/1.5% potassium ferricyanide (BDH chemicals, UK) in deionized water for 2 days at 4°C. Scaffolds were rinsed with deionized water and dehydrated in a graded series of ethanol (30/50/70/95/100 and 100% dry ethanol). They were then critically point dried using a Quorum E3100 critical point dryer (Quorum Technologies, UK) using four to five flushes with liquid CO_2_ and at least 15 min incubation between each flush. Samples were mounted on aluminium SEM stubs using conductive carbon sticky pads (Agar Scientific, UK), the sides were painted with silver DAG (TAAB, UK) for conductivity and the sample was sputter-coated with 35 nm gold, followed by 15 nm iridium using an EmiTech K575X sputter coater (Quorum Technologies, UK). Samples were viewed using an FEI Verios 460 scanning electron microscope (FEI/Thermo Fisher Scientific) run at 2.0 keV and 25 pA probe current. Secondary electron images were acquired using either an Everhart–Thornley detector in field-free mode or a through-lens detector in full immersion mode.

### 2.9. Flow cytometry

Cells were analysed with flow cytometry to demonstrate easy removal from the scaffolds and that there are separate epithelial and stromal populations, and to determine cell viability. Cells were washed with PBS then removed from the scaffolds with the addition of 1 ml of 0.2% trypsin and incubated for 5 min at 37°C. A P1000 pipette was then used to help dissociate cells by gently pipetting trypsin up and down, onto the scaffold several times before being transferred to a 15 ml Falcon tube containing 250 µl of charcoal-stripped FBS. This was repeated once more, to maximize the number of cells removed from the scaffold. Advanced DMEM/F-12 was then added to the Falcon tubes and trypsin removed by centrifugation. Cells were then transferred to a 96 v-bottom well plate and washed with FACS solution (PBS supplemented with 2% (v/v) FBS and 2 mM EDTA). Cells were stained with a live/dead marker (L10119, Thermo Fisher Scientific) and EPCAM conjugated to Alexa Flour 488 (5198, Cell Signalling, USA) in FACS solution for 30 min at room temperature and washed several times in FACS solution. An isotype control for EPCAM (550616, BD, USA) was used to help determine positive staining. To fix, 1% (v/v) PFA in PBS was added for 10 min at room temperature. Cells were then washed several times in FACS solution before acquisition on a Cytek Aurora Flow Cytometer (Cytek, USA).

### 2.10. Statistics

Data were checked for normality and homoscedasticity using the Shapiro–Wilk test and the Brown–Forsythe test, respectively, for an *α*-value of 0.05. If both tests were passed, an ordinary one-way analysis of variance and Tukey's multiple comparisons *post hoc* test were used to test for differences between groups. If either normality or homoscedasticity were not met, a Kruskal–Wallis test and Dunn's *post hoc* multiple comparisons test were used instead. The differences between groups were considered statistically significant for *p*-values less than 0.05. Statistical analysis and data plotting were carried out using Prism, v. 7 (GraphPad, USA).

## 3. Results

### 3.1. Scaffold optimization for stromal cell seeding

**Figure 2.**
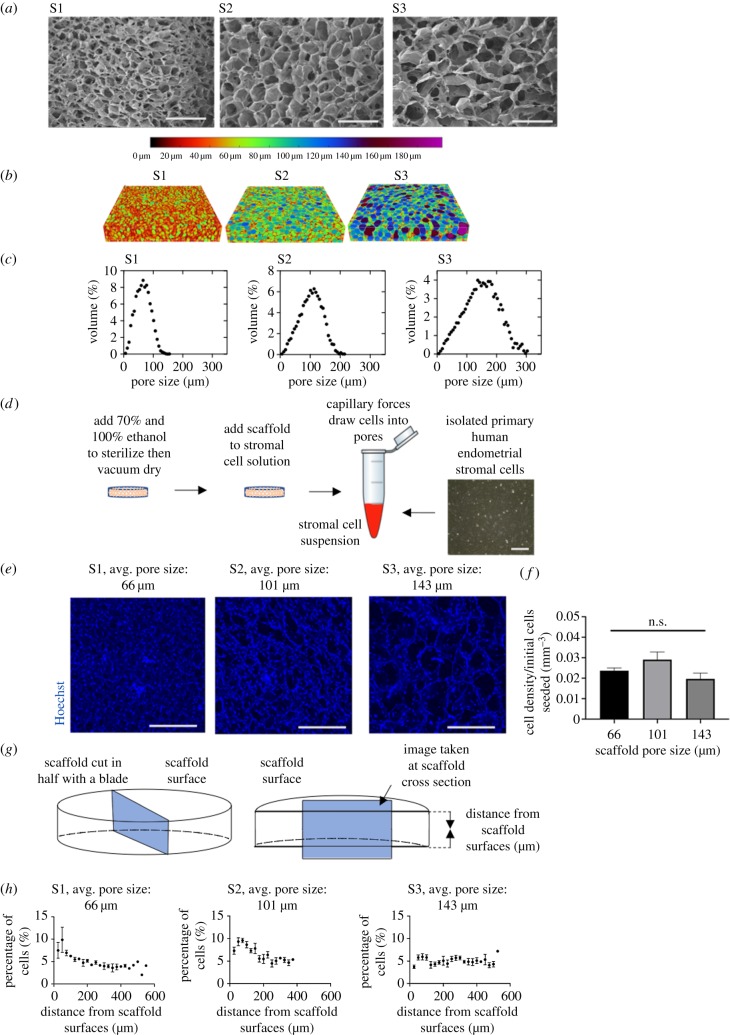
Optimization of scaffold pore size. (*a*) SEM images of the surface morphology of the pores within the collagen scaffolds. Three scaffolds of varying pore sizes were made using the following moulds: scaffold 1 (S1), a 316L stainless steel cylindrical well with a diameter of 46.5 mm, scaffold 2 (S2), a polystyrene 48-well cell culture plate, and scaffold 3 (S3), a polystyrene 6-well cell culture plate. Scale bar, 400 µm. (*b*) Micro-CT visualization of pore structures within scaffolds, with dimensions of 4 mm × 4 mm × 750 µm. (*c*) Pore size distributions based on micro-CT data. (*d*) Schematic of the seeding of primary stromal cells onto the scaffolds. Scale bar, 200 µm. (*e*) Representative images of Hoechst nuclear stain of stromal cells on the top 100 µm of scaffolds with average pore sizes of 66, 101 and 143 µm and percolation diameters of 80, 104 and 136 µm. Images were taken 7 days after seeding 183 k cells per scaffold. Scale bar, 500 µm. (*f*) Stromal cell density at the top 100 µm of the scaffold surfaces. (*g*) Schematic showing cross-section taken of scaffold. (*h*) Stromal cell location throughout the cross-section of each scaffold with average pore sizes of 66, 101 and 143 µm and percolation diameters of 80, 104 and 136 µm. Measurements were taken 7 days after seeding 332 k cells per scaffold.

In order to determine the optimal scaffold pore structure for endometrial stromal cells, scaffolds of three varying architectures were fabricated and characterized. Scaffolds 1, 2 and 3 correspond to the numbers of the moulds in which they were fabricated: S1, a 316L stainless steel cylindrical well with a diameter of 46.5 mm, S2, a polystyrene 48-well cell culture plate, and S3, a polystyrene 6-well cell culture plate. Cross-sections taken from the centre of the scaffolds were imaged with SEM to compare the relative pore sizes of the scaffolds, and micro-CT was used for a quantitative analysis of the pore structure throughout the scaffolds (figure 2*a,b*). The pore sizes showed variation between the scaffolds and broadly Gaussian distributions of pores, with pore size distributions of 66 ± 24 µm, 101 ± 38 µm and 143 ± 53 µm for scaffolds S1, S2 and S3, respectively (figure 2*c*). The average pore size for each scaffold was reproducible with a standard deviation of approximately ±10 µm between replicate samples. The percolation diameters, a characteristic of scaffold structure that gives insight into the ability of cells to travel through the scaffold, were 80 ± 11 µm, 104 ± 4 µm and 136 ± 52 µm, respectively.

Endometrial stromal cells were seeded to determine the effect of scaffold pore size on the stromal cell density and location within the scaffolds. They were seeded on scaffolds cut to a thickness of 750 µm, determined to be the minimum size for structural stability while maintaining optical clarity. After sterilization, scaffolds were vacuum-dried and added to a suspension of endometrial stromal cells. Capillary forces draw cells more deeply into the scaffolds compared with seeding on the surface (figure 2*d*). After 7 days of culture, the density of the stromal cells at the scaffold surfaces and the distribution of cells throughout its thickness were quantified by immunofluorescence. Stromal cells align with the pore architecture on the surface of all three scaffolds (figure 2*e*). Stromal cell density was determined by counting positive nuclear staining at both surfaces. It was highest for S2 with a pore size of 101 µm, although this was not statistically significant (figure 2*f*).

For the distribution of stromal cells, a cross-section through the centre of each scaffold was taken and cells were counted (figure 2*g*). In each of the scaffold types, stromal cells were found throughout the cross-section, indicating that they are able to penetrate to the centre of the scaffold. Scaffolds with the largest pore size had the most even distribution, which was expected, given that they had the largest percolation diameter (figure 2*h*). However, the large pores make the scaffold slices difficult to handle and resulted in greater distances for cells to bridge across and interconnect. Scaffold 2, with a pore size of 101 µm, was chosen for endometrial stromal three-dimensional culture, as it had the greatest overall cell density at the scaffold surface close to the luminal epithelium and was structurally stable.

### 3.2. Characterization of stromal cells cultured within the scaffold

**Figure 3.**
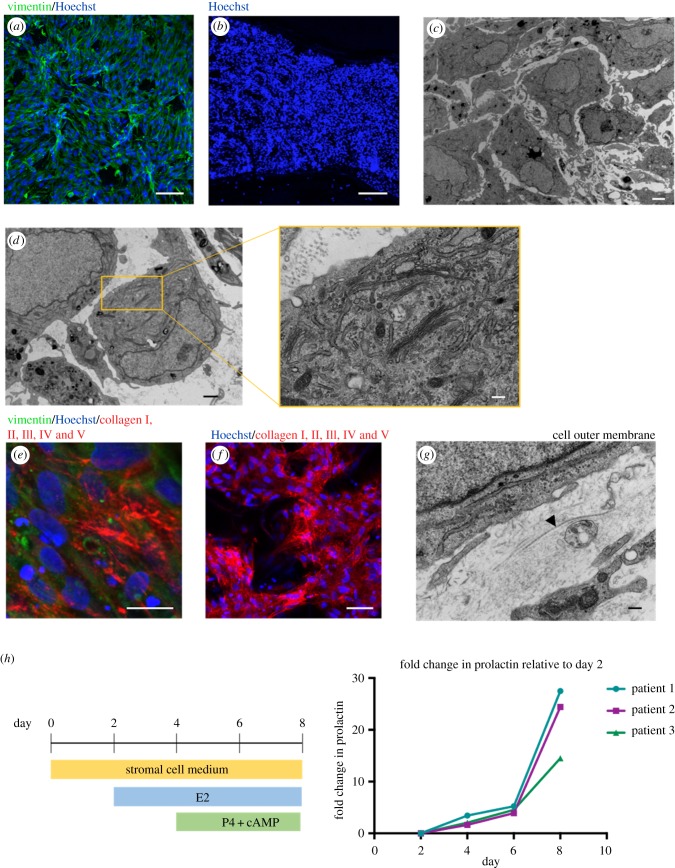
Primary endometrial stromal cells within scaffold. (*a*) Immunofluorescence of stromal cells stained with vimentin (green) and Hoechst nuclear stain (blue) at scaffold surface. Scale bar, 100 µm. (*b*) Cross-section of scaffold after 7 days of stromal cell culture, labelled with Hoechst nuclear stain (blue). Scale bar, 200 µm. (*c*) TEM of stromal cells within scaffold. Scale bar, 2 µm. (*d*) TEM showing cytoplasm containing plentiful Golgi bodies and secretion of ECM protein. Scale bar, 1 µm with inset: 200 nm. (*e*) Immunofluorescence of stromal cells showing secretion of human collagen I, II, III, IV and V (red) with vimentin (green) and Hoechst nuclear stain (blue). Scale bar, 20 µm. (*f*) Low-power immunofluorescence image, showing stromal cells are interconnected on the scaffold surface with secretions of human collagen I, II, III, IV and V (red) with Hoechst nuclear stain (blue). Scale bar, 50 µm. (*g*) TEM showing the formation of collagen bundles by stroma (see arrowhead). Scale bar, 200 nm. (*h*) Stromal cells are differentiated over 8 days within the scaffold with the addition of hormones resulting in upregulation of prolactin, measured by ELISA.

To generate a three-dimensional model of the endometrium, with stromal cells at the surface and present throughout the depth of the scaffold, a minimum seeding density of 500 k cells per scaffold was needed. This architecture is shown in figure 3*a*, with endometrial stromal cells at the scaffold surface stained with vimentin, a mesenchymal cell marker and figure 3*b*, a cross-section stained with the nuclear marker, Hoechst. Electron microscopy suggests that stromal cells cluster and proliferate in pockets within the collagen scaffold (figure 3*c*). A number of stromal cells appear bloated with plentiful Golgi apparatus and endoplasmic reticulum within each cell (figure 3*d*).

A hallmark of good *in vitro* tissue generation is deposition of a cell's own network of ECM proteins. Both endometrial and decidual stromal cells produce a wide variety of ECM components *in vivo* that includes collagens, basement membrane proteins and proteoglycans [30,31]. Endometrial stromal cells within the scaffold were positive when stained with an antibody against human collagens I, II, III, IV and V by immunofluorescence (figure 3*e*,*f*). TEM confirms the formation of collagen bundles surrounding the stromal cell membranes (figure 3*g*).

At the onset of progesterone stimulation at day 14 of the menstrual cycle, stromal cells begin to decidualize and become secretory. Stromal cells cultured within the scaffold were hormone stimulated with E2 at day 2, then P4 and cAMP, from day 4. cAMP is a secondary messenger that is commonly used in protocols to enhance differentiation. This treatment resulted in an upregulation of prolactin measured by ELISA on the culture medium, with a 14–28-fold change at day 8 relative to levels at day 2 from stromal cells derived from three different patient samples (figure 3*h*).

### 3.3. A luminal-like epithelial layer can be generated on the scaffold surface

**Figure 4.**
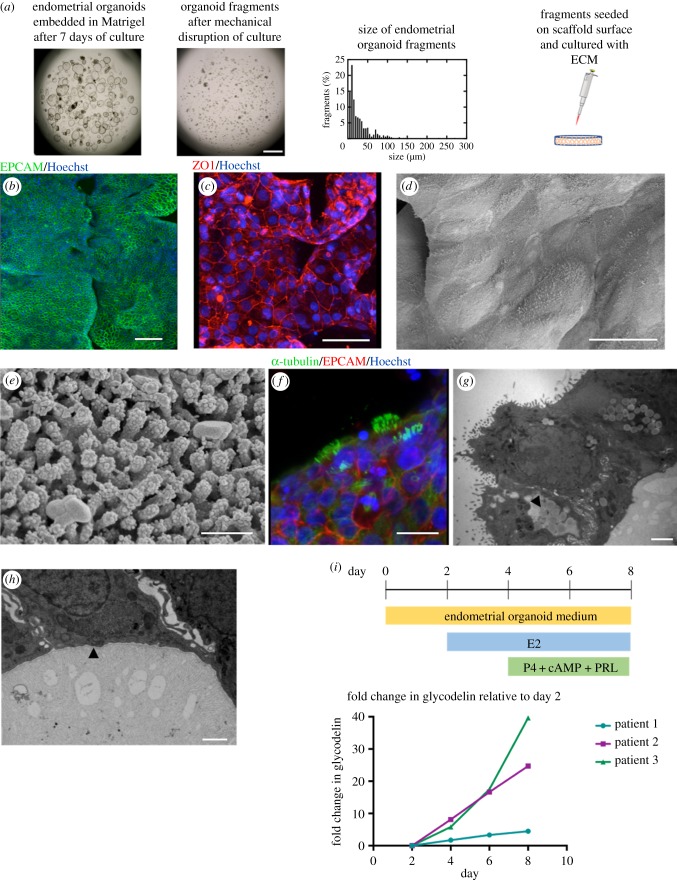
Endometrial organoid fragments seeded form a luminal-like epithelium on scaffold surface. (*a*) Endometrial organoids emended in Matrigel® are broken up mechanically to form fragments, the majority of which are less than 50 µm. These fragments are seeded on the surface of the collagen scaffold. Scale bar, 1 mm. (*b*) Immunofluorescence image of the epithelial marker EPCAM (green) on the scaffold surface with Hoechst nuclear stain (blue). Scale bar, 100 µm. (*c*) Epithelial cells form tight junctions with positive staining of Zonula occludens-1 (ZO-1, red) with Hoechst nuclear stain (blue). Scale bar, 50 µm. (*d*) SEM image of epithelial cells on scaffold surface with visible cell boundaries. Scale bar, 20 µm. (*e*) SEM showing microvilli confirming apical polarization of epithelial cells to the outside surface. Scale bar, 500 nm. (*f*) Some epithelial cells are ciliated on the scaffold surface, with staining of α-tubulin (green), EPCAM (red) and Hoechst nuclear stain (blue). Scale bar, 15 µm. (*g*) TEM shows apical polarization to the outside surface and presence of glycocalyx and lipid droplets (see arrowhead). Scale bar, 2 µm. (*h*) TEM showing a thin and fibrous lining at the basal end of the epithelial cells, indicating the formation of a basement membrane (see arrowhead). Scale bar, 1 µm. (*i*) Stimulation of epithelial cells with hormones results in increased production of glycodelin, measured by ELISA.

In order to generate an epithelium that is polarized with an exposed apical surface, EO were seeded on the scaffold surface. EO fragments were generated by mechanical disruption. The fragments should be less than 50 µm (figure 4*a*) as organoids that remain intact also adhere to the scaffold but retain their spherical shape, making them unable to form an epithelial sheet.

Organoids from two Matrigel^®^ droplets that were confluent after 7–10 days of culture were seeded per 0.5 cm^2^ of scaffold. Fragments were seeded in the centre of each scaffold and this is where confluency is greatest during culture. They become interconnected with cell outgrowth occupying the space between the fragments, appearing both as flat sheets and three-dimensional in places (figure 4*b*).

Importantly, these cells retain characteristics of luminal epithelium on the surface of the scaffold. They were able to form an epithelial barrier, confirmed with positive staining for the tight-junction protein, zonula occludens-1 (ZO-1) (figure 4*c*). SEM allows for visualization at the scaffold surface where the boundaries of individual cells (figure 4*d*) and presence of microvilli can be identified, as reported *in vivo* [4] (figure 4*e*).

The epithelial cells can be stimulated to develop cilia, a signature of uterine luminal epithelium, after differentiation with progesterone and prolactin (figure 4*f*). TEM reveals an apical basal polarization, with microvilli at the surface and fibrous material secreted underneath the cells (figure 4*g*). There are indications that these cells are laying down basement membrane, as a thin and fibrous lining is visible on the internal surface (see arrowhead, figure 4*h*), with some linearity on the matrix proteins underneath. In addition, TEM confirms the formation of tight junctions and presence of glycocalyx and lipid droplets (see arrowhead, figure 4*g*). The presence of a glycocalyx and lipid droplets at the apical surface further confirms correct polarity of the epithelial cells, and functional secretory activity.

The secretory activity of epithelial cells on the scaffold was determined with ELISA after stimulation with progesterone and prolactin. Glycodelin is a glycoprotein that plays an important role in placental development and its protein levels were found to increase by between 5 and 40 folds at day 8 relative to levels at day 2, from EO derived from three patient samples (figure 4*i*). Variability in response to hormone stimulation is expected, given the organoids are derived from primary tissue samples.

### 3.4. Co-culture of stromal and epithelial cells on the scaffold

**Figure 5.**
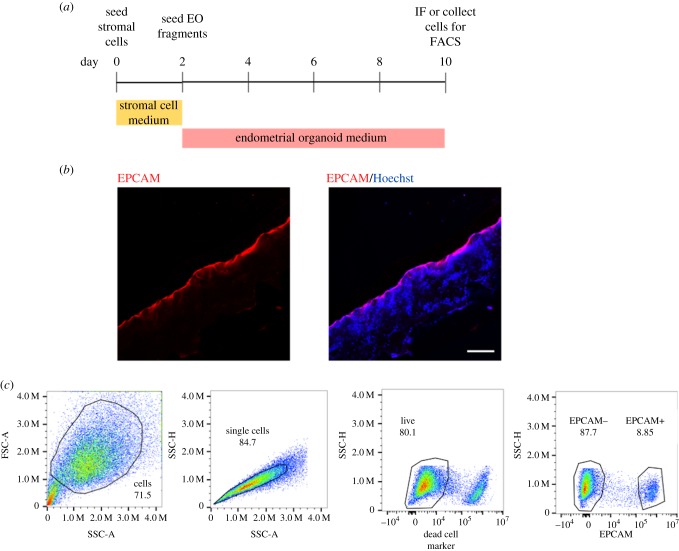
Co-culture of stromal and epithelial cells on scaffold. (*a*) Timeline of co-culture with stromal cells added at day 0, then seeding of organoid fragments at day 2. (*b*) Immunofluorescence of scaffold cross-section with epithelial cells stained with EPCAM (red) and stromal cells underneath with Hoechst nuclear stain (blue). Scale bar, 200 µm. (*c*) Cells can be removed from the scaffold for downstream analysis. Here, they were analysed using flow cytometry, showing a population of EPCAM+ (epithelial) and EPCAM– (stromal) cells.

For the co-culture of stromal and epithelial cells on the scaffold, stromal cells were seeded first and allowed to proliferate for 2 days, following the set-up described in §2.4. EO fragments were subsequently seeded, as described above and the medium changed to organoid expansion medium, to enable epithelial proliferation (figure 5*a*). Immunofluorescence was used to show both cell types on the scaffold after 10 days of co-culture, with a cross-section through the centre of the scaffold. EPCAM-positive epithelial cells line the surface with stromal cells below stained with the Hoechst nuclear marker (figure 5*b*). Both cells types can be removed from the scaffold after co-culture and used for downstream analysis. Here, we used trypsin to remove the cells and flow cytometry to confirm an EPCAM+ and EPCAM− population, for epithelial and stromal cells, respectively.

## 4. Discussion

In this study, porous collagen scaffolds have been used to develop a multi-cellular model of the endometrium that builds upon the recently established EOs. The model combines primary stromal cells with the organoids, with the aim to build the basis for a more complex model of the endometrium.

We have used porous collagen scaffolds, produced by lyophilization because they are easy to fabricate, reproducible, structurally robust for long-term culture, have good optical clarity, excellent biocompatibility and can be manipulated for different biological applications. We have taken a systematic approach to determine the optimum scaffold architecture for stromal and organoid culture. A scaffold thickness of 750 µm was determined to have the minimum thickness needed for ease of handling while maintaining optical clarity at its surface. We tested scaffolds of different pore sizes and found a pore size of 101 µm was optimal, with the highest cell density at the scaffold surface, which is important for crosstalk with the luminal epithelium. All scaffolds tested had penetration of stromal cells through the entire thickness; however, there was a reduction in cell density with depth for the smaller pore sizes. Therefore, further optimization is needed to improve stromal cell density, with one option being the development of a bioreactor system for improved nutrient and oxygen exchange.

The simplest option in the development of a co-culture model of the endometrium may be to embed stromal cells in a gel droplet with organoids; however, this does not properly model the architecture of the endometrium. Organoids embedded in Matrigel^®^ will sense ECM proteins and treat it as their basement membrane and organize themselves into spherical structures, with an apical polarization containing microvilli and ciliated cells facing inwards. A requirement for a bioengineered model is to have an exposed apical polarization of epithelial cells, in order to begin to replicate the uterine luminal epithelium. When organoid fragments are seeded on the scaffold, without the physical confinement of a gel, they lose their spherical structure and begin to proliferate. While not every cell is in contact with the collagen scaffold, all cells will polarize with their apical side facing the outside surface. We confirmed apical polarization to the outside surface with TEM, SEM and immunofluorescence.

This study moves beyond using the artificial ECM, Matrigel^®^, which has batch-to-batch variation in mechanics and protein content and instead provides a scaffold to enable both stromal and epithelial cells to deposit their own ECM networks, that include collagen bundles [32]. Together with matrix protein deposition, another hallmark of a good bioengineered model is functionality of the cells *in vitro*. Here, we hormone stimulated both cell types individually on the scaffold and this resulted in production of secretory proteins that are associated with epithelial differentiation and stromal decidualization. The next step is to manipulate the architecture of the scaffold to direct the organoid fragments to organize into glands, which are the secretory epithelial population of the endometrium.

We have taken the first steps to co-culture epithelial and stromal cells on the scaffold. Stromal cells are seeded first and allowed to proliferate for 2 days, and organoid fragments are seeded on the scaffold surface to enable the formation of a luminal-like epithelial layer. Both cell types can be easily removed from the scaffold for downstream analysis, which was demonstrated by flow cytometry. This is important if the model is to be used to study stromal–epithelial interactions and to use techniques to quantify gene and protein expression.

## 5. Summary

We have built upon the recently established EOs and have developed a porous collagen scaffold-based model of the endometrium containing both epithelial and stromal cells. A systematic approach was taken to determine the optimum scaffold pore size for stromal cell culture, and this was found to be 101 µm. EOs seeded organize to form a luminal-like epithelial layer on the scaffold, with apical polarization towards the outside surface. Importantly, both stromal and epithelial cells are functionally responsive to hormones on the scaffold. The next step is to manipulate the architecture of the scaffold in order to form gland-like structures and integrate both immune and trophoblast cells.

## Data accessibility

Supporting research data including raw data used to optimize collagen scaffolds for primary endometrial stromal cell seeding may be accessed at: https://doi.org/10.17863/CAM.41681.
